# Impact of pump position on postoperative outcomes in less invasive left ventricular assist device implantation

**DOI:** 10.3389/fcvm.2025.1591653

**Published:** 2025-08-15

**Authors:** Tomoyuki Suzuki, Hayato Ise, Robin Döpp, Patric Kröpil, Yoshikatsu Saiki, Artur Lichtenberg, Udo Boeken, Hug Aubin, Payam Akhyari, Yukiharu Sugimura

**Affiliations:** ^1^ Department of Cardiac Surgery and Research Group for Experimental Surgery, Medical Faculty and University Hospital Düsseldorf, Heinrich-Heine-University, Düsseldorf, Germany; ^2^ Division of Cardiovascular Surgery, Tohoku University Graduate School of Medicine, Sendai, Japan; ^3^ Department of Radiology, BG Klinikum Duisburg, Duisburg, Germany; ^4^ Department of Diagnostic and Interventional Radiology, Medical Faculty, University of Dusseldorf, Dusseldorf, Germany

**Keywords:** left ventricular assist device (LVAD), pump position, less invasive approach (LIS), cannula coronal angle (CCA), pump diaphragm depth (PDD)

## Abstract

**Introduction:**

less invasive approach (LIS) has recently become increasingly used for left ventricular assist device (LVAD) implantation. However, the impact of surgical access on pump position and clinical outcomes comparing LIS-LVAD implantation to full sternotomy (ST) has not been well discussed.

**Methods:**

Between April 2010 and February 2021, a total of 237 consecutive patients received a LVAD, 76 (32.1%) of whom underwent the LIS approach and 161 (67.9%) of whom underwent ST. The clinical outcomes were retrospectively reviewed, and data of 66 comparable patients from each group extracted by propensity score matching were analyzed. For the analysis of pump position, cannula coronal angle (CCA,°) and pump diaphragm depth (PDD, mm) of LVAD were measured according to postoperative chest x-ray.

**Results:**

The mean age of all patients was 57.7 ± 11.3 years, 204 cases were male (86.1%), and 48 cases resulted in in-hospital death (20.3%). There was no significant impact on clinical outcomes according to surgical approach in matched groups. There was also no significant difference regarding pump position between two different access groups. A larger PDD was associated with both in-hospital death (60.2 ± 25.8 vs. 43.4 ± 31.3, *P* < 0.01) and death on LVAD (55.4 ± 28.1 vs. 41.7 ± 31.5, *P* < 0.01). Receiver operating characteristic (ROC) curve analyses revealed that PDD was a significant predictor of mortality in LIS approach.

**Conclusions:**

Our results indicate that LVAD implantation via LIS approach is safe yielding comparable outcomes with ST approach. Regarding spatial positioning of LVAD via LIS approach, larger PDD, may predict worse clinical outcomes.

## 1 Introduction

The 13th annual report from The Society of Thoracic Surgeons (STS) Interagency Registry for Mechanically Assisted Circulatory Support (INTERMACS) includes more than 2,400 left ventricular assist device (LVAD) implantations in 2021 and more than 80% of them performed as destination therapy (1). Even when LVADs are used as a bridge to transplantation, it is important to keep patients in good condition during the relatively long waiting period for transplantation. Thus, the importance of long-term management of LVAD patients in maintaining a stable condition can never be overemphasized. To achieve this goal, meticulous managements including prevention of right heart failure exacerbation (2), Anticoagulation (3) or infection preventing measures (4) are essential. Recently, pump position has been suggested as a determinant of clinical outcome, specifically, the in-flow angle and pump depth have been noted as significant positional parameters (5–8). Besides patient anatomy, surgical procedure and access may also be affecting postoperative pump position (9, 10). As a relatively new technique, the clinical outcomes after LVAD implantation with less invasive approach (LIS) and the impact of this method on postoperative pump position remain uncharacterized. Hence, as a primary proposition, we examined and compared clinical outcomes of the patients with LIS vs. sternotomy (ST) together with pump position as potential confounding factors. Further, pump position was analyzed to clarify how positional factors relate to outcome and whether there is a surgical access-related difference.

## 2 Materials and methods

### 2.1 Ethics

The investigation conforms with the principles outlined in the Declaration of Helsinki and was approved by the local ethics committee in university hospital Düsseldorf (2020-1058).

### 2.2 Study design

Between April 2010 and February 2021, a total of 247 consecutive patients received a LVAD in university hospital Düsseldorf. Ten cases with HeartmateⅡ were excluded from analysis, because this device has a design that is considerably larger than HeartMate 3 or Heartware, and it has been introduced in the early millennium years and represents a previous generation with potentially increased risk of pump thrombosis or thromboembolic events. For the remaining cases, Heartware (*n* = 159, 67.1%) or HeartMate 3 (*n* = 78, 32.9%) were implanted. Of those, LIS was performed in 76 cases (32.1%) while median full sternotomy (ST) was performed in 161 cases (67.9%). Propensity score matching was performed to align the characteristics between groups and to make precise analyses. Cannula coronal angle (CCA) and pump diaphragm depth (PDD) of LVAD were measured by assessing postoperative x-ray. The clinical outcomes were retrospectively reviewed and analyzed by comparing the applied two distinct surgical approaches, i.e., LIS vs. ST in relation to the postoperative LVAD position.

### 2.3 LIS approach

The actual maneuver has been described previously (11). As a major incision, partial upper (“J” shaped) sternotomy is performed between the first and fourth intercostal space. Aortic cannulation to distal ascending aorta and percutaneous femoral vein cannulation with percutaneous vascular closure device application (ProGlide; Abbott, Abbott Park, IL) are established for cardio-pulmonary bypass after systemic heparinization. Simultaneously, a left anterolateral mini-thoracotomy is performed at fifth intercostal space. After incising the apex of the left ventricle, the inflow component is inserted through the ventriculotomy and a centrifugal pump is fixed on the sewing ring. For Heartware and HeartMate 3 the instructions for users from the manufacturer are followed. The driveline is externalized in a “C”-shaped fashion. Outflow graft is tunneled intra-pericardially toward the ascending aorta and anastomosed to the anterolateral aspect of the aorta using standard side clamping technique or using a proximal anastomotic seal device (Heart String; Getinge, Göteborg, Sweden) (12).

### 2.4 Evaluation of LVAD positioning

**Figure 1:**
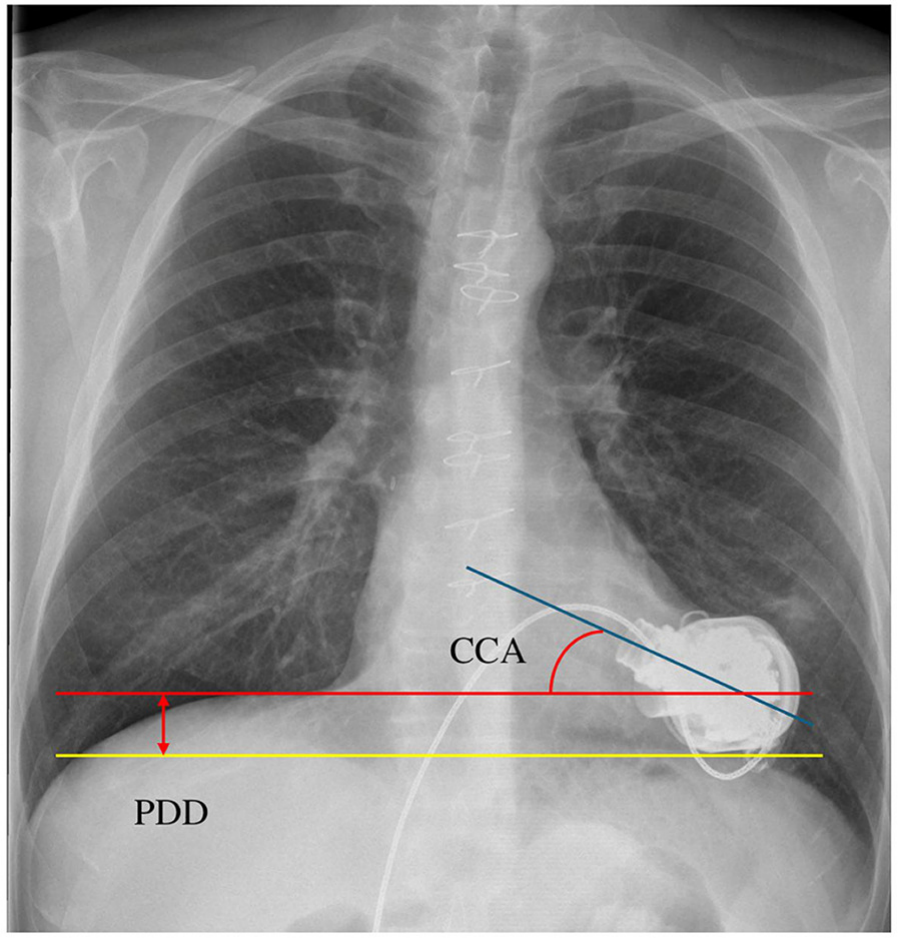
The actual image in measurement of CCA and PDD. CCA, cannula coronal angle; PDD, pump diaphragm depth.

The spatial parameters of LVAD position were measured on x-ray images 1 month after the operation. The following algorithm was used for each measurement, according to descriptions in previous literature as to angle (5, 6) and depth (6, 8). The angle of the cannula axis relative to a horizontal line was measured as CCA. The height between the top of right diaphragm and the bottom line of pump body was measured as PDD. A representative image of measurement is shown in Figure 1.

### 2.5 Statistics

Chi-square tests were used for comparisons of categorical data between groups, and independent-samples *t*-test was used for comparisons of means of continuous variables. All these statistical method and analysis including propensity score matching and receiver operating characteristic curve analysis were performed using IBM SPSS Statistics version 28 (IBM Corp, Armonk, NY) and the level of statistical significance was set at *p* < 0.05. Age, sex, BMI, LVAD type (Heartware or HeartMate 3), preoperative impaired right ventricular function and INTERMACS score were included as factors for propensity score calculation. Caliper for matching was set as 0.20 times the standard deviation of the propensity score. The balance in each characteristic between matched groups were checked with standardized mean difference (SMD). SMDs for most characteristics were 0.1 or less except for sex (0.20), dilated cardiomyopathy (0.29) and ischemic cardiomyopathy (0.22) (Supplementary Table 1). SMD calculation and violin plot were performed with EZR (Jichi Medical University, Tochigi, Japan), which is a graphical user interface for R (The R Foundation for Statistical Computing, Vienna, Austria).

## 3 Results

### 3.1 Baseline characteristics in all cohorts

The characteristics of all 237 patients and each group are shown in Table 1. Mean age was 57.7 ± 11.3 years, and 204 (86.1%) patients were male. The ratio of the patients with INTERMACS score ≦2.0 was significantly higher in Group S (69.6% vs. 31.6%). By propensity score matching, 66 patients were extracted from each group, resulting in the total number of 132 patients entered in the final analysis. Baseline characteristics in matched groups are shown in Supplementary Table 1.

**Table 1:** Patient characteristics in overall cohort.

Variables	All (*n* = 237)	ST (*n* = 161)	LIS (*n* = 76)	*P*
Age (y)	57.7 ± 11.3	56.4 ± 11.8	60.3 ± 9.8	0.01
Male, *n* (%)	204 (86.1)	137/161 (85.1)	67/76 (88.2)	0.53
Height (m)	1.76 ± 0.08	1.75 ± 0.09	1.77 ± 0.08	0.33
Weight (kg)	83.7 ± 18.4	82.8 ± 18.8	85.7 ± 17.3	0.25
BMI	27.0 ± 5.5	26.9 ± 5.7	27.4 ± 5.2	0.49
Dialysis, *n* (%)	46 (19.4)	35/161 (21.7)	11/76 (14.5)	0.19
DCM, *n* (%)	87 (36.7)	56/161 (34.8)	31/76 (40.8)	0.37
ICM, *n* (%)	146 (61.6)	101/161 (62.7)	45/76 (59.2)	0.60
Heartware:HM3, n:n (%:%)	159:78 (67.1:32.9)	114:47 (70.8:29.2)	45:31 (59.2:40.8)	0.08
Impaired RV function, *n* (%)	91 (38.4)	64/161 (39.8)	27/76 (35.5)	0.53
INTERMACS score ≦2, *n* (%)	136 (57.4)	112 (69.6)	24 (31.6)	<0.01

Data documented as *n* (%) or mean ± standard deviation. ST, group ST (sternotomy); LIS, group LIS (less invasive approach), BMI, body mass index; DCM, dilated cardiomyopathy; ICM, ischemic cardiomyopathy; HM3, HeartMate 3; RV, right ventricle; INTERMACS, Interagency Registry for Mechanically Assisted Circulatory Support.

### 3.2 Analysis of clinical outcomes according to surgical approach

**Figure 2:**
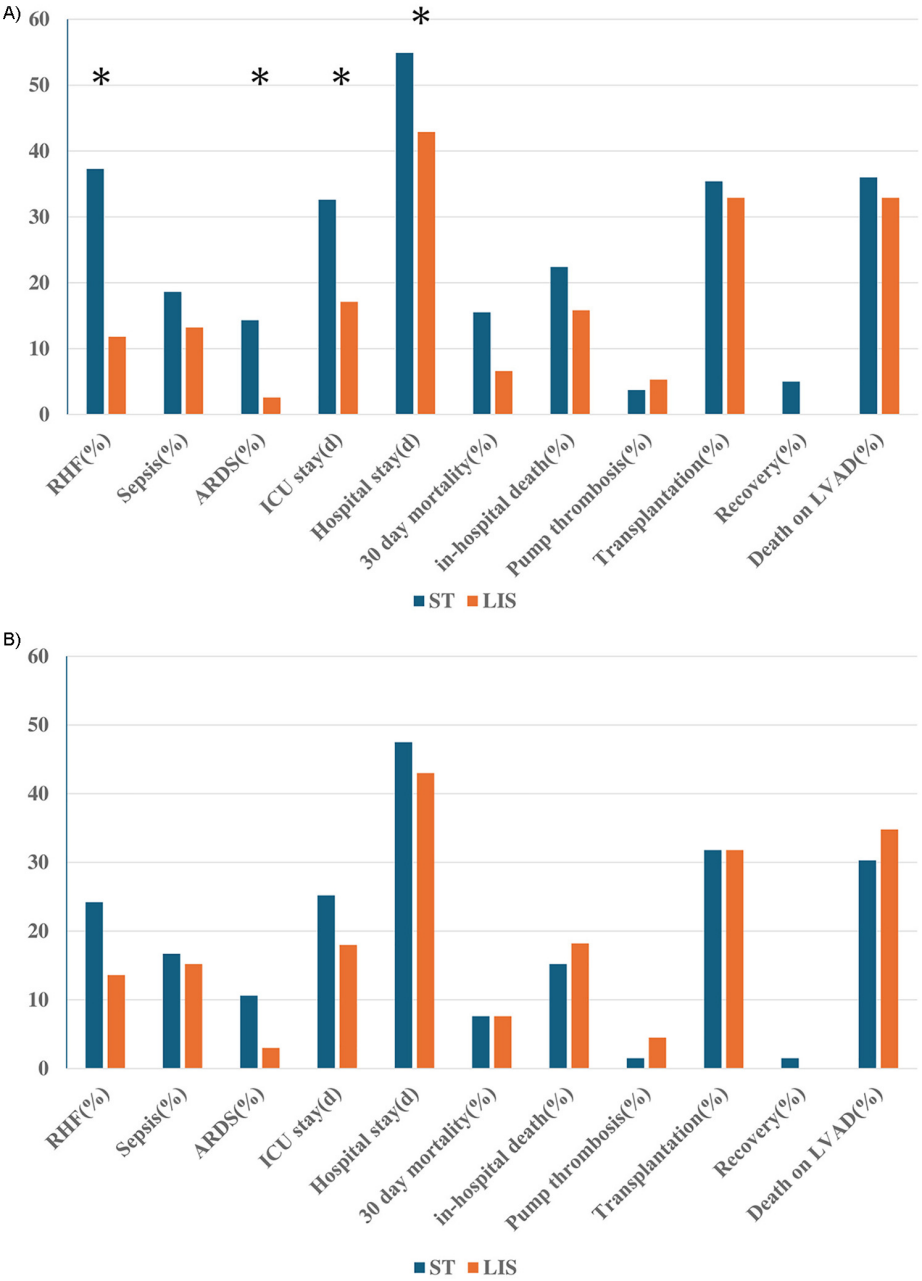
Clinical outcome in ST and LIS from allover cohort **(A)** and matched groups **(B)** the difference between groups under *p*-value of 0.05 is shown with*. RHF, right heart failure; ARDS, acute respiratory distress syndrome; ICU, intensive care unit; LVAD, left ventricular assist device.

Clinical outcomes of the unmatched groups are shown in Supplementary Table 2. There were 48 (20.3%) in-hospital deaths (2 patients died after transplantation), and 83 cases (35.0%) of death during all support period including in-hospital stay. Eighty two patients (34.6%) were successfully bridged to heart transplantation with a mean waiting time of 447.8 ± 429.7 days. Among all cohorts, LIS patients achieved significantly favorable outcomes in several categories (right heart failure, Acute respiratory distress syndrome, ICU stay, Hospital stay). After propensity score matching, there were no differences in clinical outcomes between ST and LIS cohorts, suggesting similar quality and safety with LIS approach based on the analyzed parameters (Table 2). The clinical outcomes in each group from overall cohort and matched cohort are shown as graph in Figures 2A,B.

**Table 2:** Clinical outcome in matched groups.

Outcomes	All (*n* = 132)	ST (*n* = 66)	LIS (*n* = 66)	*P*
Right heart failure, *n* (%)	25 (18.9)	16 (24.2)	9 (13.6)	0.12
Sepsis, *n* (%)	21 (15.9)	11 (16.7)	10 (15.2)	0.81
Acute respiratory distress syndrome, *n* (%)	9 (6.8)	7 (10.6)	2 (3.0)	0.16
ICU stay (d)	21.6 ± 26.5	25.2 ± 24.1	18.0 ± 28.4	0.12
Hospital stay (d)	45.3 ± 36.3	47.5 ± 35.1	43.0 ± 37.5	0.47
30 day mortality, *n* (%)	10 (7.6)	5 (7.6)	5 (7.6)	1.00
In-hospital death, *n* (%)	22 (16.7)	10 (15.2)	12 (18.2)	0.64
Pump thrombosis, *n* (%)	4 (3.0)	1 (1.5)	3 (4.5)	0.62
Transplantation, *n* (%)	42 (31.8)	21 (31.8)	21 (31.8)	1.00
Recovery, *n* (%)	1 (0.8)	1 (1.5)	0 (0)	1.00
Death on LVAD, *n* (%)	43 (32.6)	20 (30.3)	23 (34.8)	0.58

Data documented as *n* (%) or mean ± standard deviation. ST, Group S (sternotomy); LIS, Group L (less invasive approach); ICU, intensive care unit; LVAD, left ventricular assist device.

### 3.3 Analysis of spatial position (CCA, PDD)

With regards to the position of LVAD in matched cohort, the mean CCA was 19.8 ± 21.1°, and mean PDD was 44.7 ± 31.9 mm. There were no significant differences in CCA and PDD between group ST and LIS (CCA: 22.6 ± 22.0° vs. 17.4 ± 20.2°, *P* = 0.19, PDD: 45.4 ± 28.1 mm vs. 44.2 ± 35.2 mm, *P* = 0.85) (Table 3). These data suggest that the LIS approach does not affect the LVAD position as compared to ST. In order to further illustrate variability and group-level trends, violin plots for CCA and PDD were also described (Supplementary Figure 1).

**Table 3:** LVAD position and surgical approach: matched groups.

Positional parameters	All (*n* = 132)	ST (*n* = 66)	LIS (*n* = 66)	*P*
CCA	19.8 ± 21.1 (*n* = 117)	22.6 ± 22.0 (*n* = 55)	17.4 ± 20.2 (*n* = 62)	0.19
PDD	44.7 ± 31.9 (*n* = 113)	45.4 ± 28.1 (*n* = 53)	44.2 ± 35.2 (*n* = 60)	0.85

Data documented mean ± standard deviation. ST, Group ST (sternotomy); LIS, Group LIS (less invasive approach); CCA, cannula coronal angle; PDD, pump diaphragm depth.

### 3.4 Impact of spatial position on mortality

When the positional parameters were compared between mortal and non-mortal groups among all patients, a significantly greater mean PDD was observed in patients with in-hospital death (in-hospital death+: 60.2 ± 25.8 mm vs. in-hospital death−: 43.4 ± 31.3 mm, *P* < 0.01) as well as in patients who died during all support period after implantation (death on LVAD+: 55.4 ± 28.1 mm vs. death on LVAD−: 41.7 ± 31.5 mm, *P* < 0.01). There was no significant difference in CCA between survivors and non-survivors (Table 4).

**Table 4:** Clinical outcome and LVAD position: overall cohort.

Positional parameters	In hospital death + (*n* = 48)	In hospital death – (*n* = 189)	*P*
CCA	22.1 ± 19.8 (*n* = 31)	20.2 ± 21.3 (*n* = 175)	0.64
PDD	60.2 ± 25.8 (*n* = 29)	43.4 ± 31.3 (*n* = 168)	<0.01
Positional parameters	Death on LVAD + (*n* = 83)	Death on LVAD – (*n* = 154)	*P*
CCA	20.6 ± 20.4 (*n* = 65)	20.5 ± 21.4 (*n* = 141)	0.98
PDD	55.4 ± 28.1 (*n* = 61)	41.7 ± 31.5 (*n* = 136)	<0.01

Data documented as *n* (%) or mean ± standard deviation. CCA, cannula coronal angle; PDD, pump diaphragm depth; LVAD, left ventricular assist device.

### 3.5 ROC analysis of CCA and PDD for mortality

**Figure 3:**
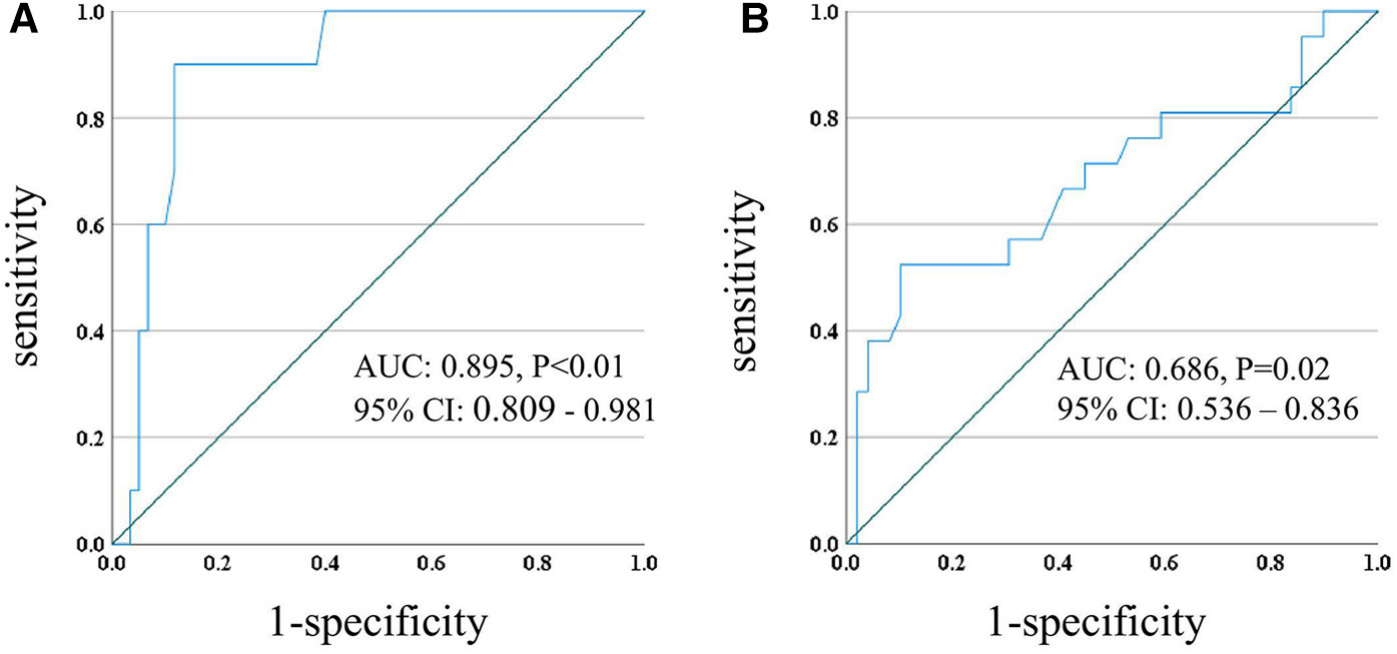
ROC curve of PDD and in-hospital mortality **(A)**, PDD and death on LVAD **(B)** in group LIS. ROC, receiver operating characteristic; PDD, pump diaphragm depth; LVAD, left ventricular assist device; AUC, area under the curve; CI, confidence intervals.

**Figure 4:**
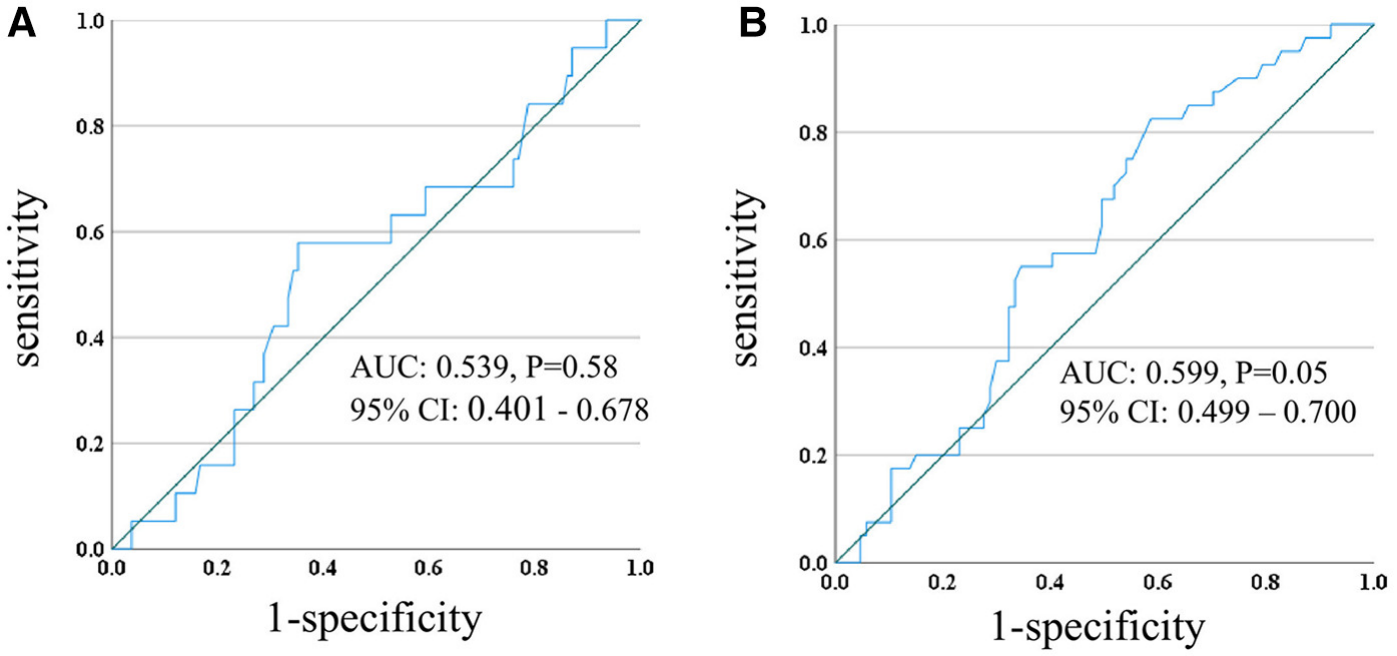
ROC curve of PDD and in-hospital mortality **(A)**, PDD and death on LVAD **(B)** in group ST. ROC, receiver operating characteristic; PDD; pump diaphragm depth, LVAD, left ventricular assist device; AUC, area under the curve; CI, confidence intervals.

Receiver operating characteristic (ROC) curve analyses revealed that PDD was a significant predictor of both in-hospital mortality (AUC: 0.674, 95% CI: 0.569–0.780, *p* < 0.01, cut-off 56.6 mm with a sensitivity of 0.690 and specificity of 0.690) and whole mortality on LVAD support (AUC: 0.634, 95% CI: 0.551–0.717, *p* < 0.01, cut-off 55.6 mm with a sensitivity of 0.541 and specificity of 0.691) in the overall cohort, while CCA had no significant effect on mortality. Upon ROC analysis, PDD was significantly predictive in LIS group both for in-hospital mortality (AUC: 0.895, 95% CI: 0.809–0.981, *p* < 0.01, cut-off 69.2 mm with a sensitivity of 0.90 and specificity of 0.88) and mortality on LVAD support (AUC: 0.686, 95% CI: 0.536–0.836, *p* = 0.02, cut-off 69.2 mm with a sensitivity of 0.52 and specificity of 0.90) (Figures 3A,B), while there was no significant predictive impact of PDD in group ST (Figures 4A,B). The number of cases with large PDD over the cut-off length of 69.2 mm was 24 (18.9%) in Group ST and 16 (22.9%) in Group LIS. There was no difference between the two groups (*p* = 0.508).

## 4 Discussion

Although the LIS approach for LVAD implantation has long been established, there is still controversy regarding comparable accuracy of pump positioning when compared to full sternotomy approach. In this study with a total of 237 LVAD implantations, we demonstrated equivalent outcomes derived from LIS and ST approach with respect to anatomical positioning of the LVAD as well as clinical end points in a propensity matched cohort analysis. Interestingly, PDD showed a statistically positive association with higher mortality in the subset of patients with LIS approach, which was not present in ST group.

Data from previous studies on the value of the LIS approach for LVAD implantation are mixed with respect to a variety of endpoints. Stable positioning of the inflow cannula, prevention of postoperative right heart failure and all-cause mortality after LVAD implantation have been the subject of a number of clinical investigations (13–17), with no homogeneous picture conveyed by the current body of evidence. Here, using a matched pair (*n* = 66 in each) analysis with ST and LIS approach, we demonstrate comparable outcomes regarding mortality and other relevant clinical endpoints. These findings underline the safety of LIS approach for LVAD implantation. We observed a relatively lower absolute number of right heart failure in LIS patients as compared to sternotomy patients (9 vs. 16), although this difference was not statistically significant. This finding is consistent with the results inferred from previous reports, which show the better RV function in LIS patients for whom the anterior aspect of pericardium is preserved (15, 16).

Next, we determined LVAD position in our patients at one month after implantation by measuring CCA and PDD. We tested the hypothesis whether LVAD position in interplay with the respective implantation approach may have an impact on clinical outcome. The underlying rationale is the categorical difference between LIS and ST with respect to preservation of the anterior pericardial continuity and also pleural involvement. Moreover, as some of patients in LIS group received a unique technique for anastomosis of the outflow graft to the aorta, one may speculate these and other technical differences collectively will impact on the clinical outcome for LIS patients when compared with those receiving ST LVAD implantation. With regard to CCA, a correlation between wide CCA and higher hemocompatibility-related adverse events has been reported for Heartware Ventricular Assist Device (5). Pump thrombosis is one of the major adverse events after LVAD implantation and may cause cerebral embolism and/or pump failure. Suboptimal inflow cannula angle or position have also been suggested as possible risk factors for intraventricular thrombosis (18–20). In our matched cohort, there were 1 case of pump thrombosis in Group ST and 3 in Group LIS, and all of these 4 patients had received Heartware devices. Inflow cannula position also relates to the capability of cardiac unloading (6) and subsequently the degree of mitral valve regurgitation (21), thus it influences the status of systemic circulation and possibly also the extent to which heart failure symptoms are modulated by LVAD therapy. CCA is an important indicator for the adequacy of cannula position and may therefore have an impact on clinical outcomes (5–8). We found that CCA had no impact on mortality in our cohort. One possible explanation is relatively lower mean CCA of approximately 20 degrees in our cohort compared to the median CCA of nearly 40 degrees reported in previous studies (5).

In contrast to CCA, we identified larger PDD as a significant risk factor for in-hospital and all mortality after LVAD implantation. Further, ROC curve analysis showed that large PDD is predictive for mortality only in group LIS. This finding suggests that a potentially negative impact of larger PDD may be exacerbated by LIS approach resulting in clinically relevant adverse outcomes. The exact mechanisms by which surgical approach may impact PDD during LVAD implantation are yet unknown. One possible mechanism by which surgical access interacts with PDD is the anterior longitudinal incision and division of the pericardial sac leading to a widening of the intrapericardial space after median sternotomy. This leads to a more horizontal expansion of the heart along with the apically positioned LVAD. This mechanism may therefore lead to smaller PDD in the full sternotomy group. On the other hand, thoracotomy approach typically is associated with prolonged discomfort during physical activity and particularly leads to thoracic pain during forced inhalation, resulting in shallow breathing activity in the early postoperative phase. This latter mechanism would rather lead to larger PDD calculated based on thoracic Xray in the first 2–4 weeks after LIS. In our series however, there was actually no significant difference in mean PDD between groups, suggesting that there may be a different impact of PDD on outcome in LIS patients. Previous literature has suggested PDD as one of predictors of heart failure or death in univariate analysis, although it has not been shown to be significant in multivariable analysis (6).

When LIS patients were divided according to PDD above or lower than the cut-off (i.e., 69.2), patients with PDD > 69.2 had significantly higher rate of impaired pre-Operative right ventricle (RV) dysfunction, significantly higher rate of post-Operative need of right ventricular assist device (RVAD), higher rate of post-Operative dialysis, and also higher rates of stroke and tracheostomy. The results of this subgroup analysis are shown in Supplementary Table 3. We interpret these findings as a support for the hypothesis that in patients with LIS RV-related complications may be accentuated when PDD becomes large. The main difference to the sternotomy group may be explained by the ventral pericardial incision in ST group, which traditionally is perceived as a detrimental factor for post-operative RV dysfunction. However, in patients with large dimensions of both ventricles the pericardial incision modulates the available space for the compartment bearing the heart and the LVAD device. In these patients, preserving the anterior part of the pericardial sac during LIS may lead to higher postoperative PDD, which in turn may increase the risk of RV-related adverse outcomes.

Notably, besides implantation technique, large PDD can result not only from anatomically deep apex, but also from higher diaphragm. We have introduced a specific LVAD implantation method with so called “Furoshiki technique” for LIS case from 2018, covering the implanted pump with both polytetrafluoroethylene (PTFE) (Gore Preclude pericardial membrane; W.L. Gore and Associates, Tempe, AZ) and bovine pericardium (11). This technique potentially has a chance of not only avoiding the severe adhesion at the later time point of heart transplantation, but also steadier pump positioning after implantation. Thus, we expect to be able to achieve a more accurate pump position in LIS technically. Cases with large PDD in LIS patients may have more significance with the height of the diaphragm than the pump position itself. Factors which make the diaphragm higher and thus increase PDD may simultaneously influence an unfavorable prognosis in LIS patients. Although further factorial analysis could not be clarified in this study, patients with large PDD should be carefully monitored, particularly when LIS approach is used.

## 5 Conclusions

LVAD implantation via LIS approach is safe yielding comparable outcomes as with ST approach, without significant differences in positioning parameters of CCA and PDD. However, in patients with LIS approach larger PDD is associated with post-operative mortality. PDD may predict worse clinical outcomes in LVAD patients via LIS.

## 6 Limitations

LVAD related adverse events, such as readmission for heart failure symptoms or hemocompatibility related adverse events, or parameters of hemodynamic status are not fully evaluated. The body position at x-ray imaging is not universally defined (spine, seated or standing), and the angle evaluation is not performed by three-dimensional imaging.

## Data Availability

The original contributions presented in the study are included in the article/Supplementary Material, further inquiries can be directed to the corresponding author.

## Supplementary material

Supplementary Figure 1 Violin plot with box plot of CCA in group ST and LIS **(A)**, PDD in group ST and LIS **(B)**: matched groups. CCA; cannula coronal angle, PDD; pump diaphragm depth, ST; sternotomy, LIS; less invasive approach.
