# Supplementary figures and images for: Impact of pump position on postoperative outcomes in less invasive left ventricular assist device implantation
Source: Front Cardiovasc Med. 2025 Aug 15;12:1591653. doi: 10.3389/fcvm.2025.1591653 (PMC12394547; doi:10.3389/fcvm.2025.1591653)

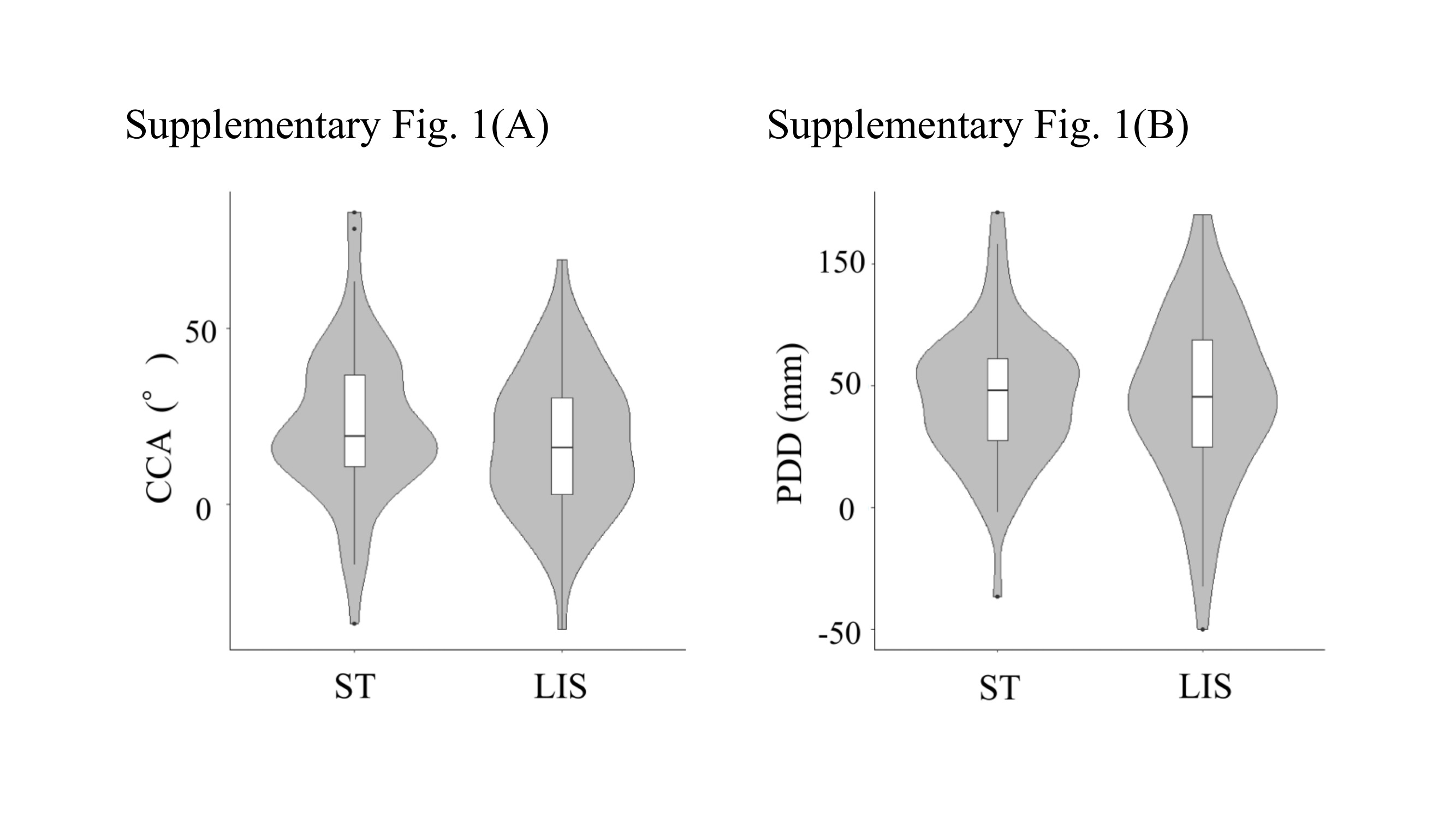

Supplement: Supplementary Figure 1 — Violin plot with box plot of CCA in group ST and LIS (A), PDD in group ST and LIS (B): matched groups. CCA; cannula coronal angle, PDD; pump diaphragm depth, ST; sternotomy, LIS; less invasive approach. [file Image1.tiff]
