# Supplementary material for: Impact of pump position on postoperative outcomes in less invasive left ventricular assist device implantation
Source: Front Cardiovasc Med. 2025 Aug 15;12:1591653. doi: 10.3389/fcvm.2025.1591653 (PMC12394547; doi:10.3389/fcvm.2025.1591653)
Supplement: Supplementary file 2 [file Datasheet1.pdf]

Supplementary Material

1 Supplementary Data

Supplementary Material should be uploaded separately on submission. Please include any supplementary data, figures and/or tables.

Supplementary material is not typeset so please ensure that all information is clearly presented, the appropriate caption is included in the file and not in the manuscript, and that the style conforms to the rest of the article.

2 Supplementary Figures and Tables

Supplementary Table 1 Patient characteristics in matched groups

	All (n=132)	ST (n=66)	LIS (n=66)	P	SMD
Age (y)	60.1±10.5	60.2±11.1	60.0±9.9	0.91	0.02
Male, n (%)	118 (89.4)	61 (92.4)	57 (86.4)	0.26	0.20
Height (m)	1.76±0.08	1.77±0.08	1.77±0.09	0.87	0.03
Weight (kg)	85.9±18.2	86.8±19.2	85.0±117.3	0.57	0.10
BMI	27.4±5.5	27.7±5.9	27.1±5.1	0.53	0.11
Dialysis, n (%)	19 (14.4)	9 (13.6)	10 (15.2)	0.80	0.04
DCM, n (%)	49 (30.3)	20 (34.8)	29 (43.9)	0.11	0.29
ICM, n (%)	81 (61.4)	44 (66.7)	37 (56.1)	0.21	0.22
Heartware:HM3, n:n (%:%)	76:56	38:28 (57.6:42.4%)	38:28 (57.6:42.4%)	1.00	<0.01
Impaired RV function, n (%)	52 (39.4)	27 (40.9)	25 (37.9)	0.72	0.06
INTERMACS score≦2, n (%)	48 (36.4)	25 (37.9)	23 (34.8)	0.72	0.06

Data documented as n (%) or mean ± standard deviation. ST, group ST (sternotomy); LIS, group LIS (less invasive ~~approach~~), BMI, body mass index; DCM, dilated cardiomyopathy; ICM, ischemic cardiomyopathy; HM3, Heartmate 3; RV, right ventricle; INTERMACS, Interagency Registry for Mechanically Assisted Circulatory Support; SMD, standardized mean difference.

Supplementary Table 2 Clinical outcome in all-over cohort

	All (n=237)	ST (n=161)	LIS (n=76)	P
Right heart failure, n (%)	69 (29.1)	60 (37.3)	9 (11.8)	<0.01
Sepsis, n (%)	40 (16.9)	30 (18.6)	10 (13.2)	0.29
Acute respiratory distress syndrome, n (%)	25 (10.5)	23 (14.3)	2 (2.6)	<0.01
ICU stay (d)	27.6±28.4	32.6±27.6	17.1±27.3	<0.01
Hospital stay (d)	51.1±38.8	54.9±39.6	42.9±35.9	0.03
30day mortality, n (%)	30 (12.7)	25 (15.5)	5 (6.6)	0.05
In-hospital death, n (%)	48 (20.3)	36 (22.4)	12 (15.8)	0.24
Pump thrombosis, n (%)	10 (4.2)	6 (3.7)	4 (5.3)	0.73
Transplantation, n (%)	82 (34.6)	57 (35.4)	25 (32.9)	0.71
Recovery, n (%)	8 (3.4)	8 (5)	0 (0)	0.06
Death on LVAD, n (%)	83 (35.0)	58 (36.0)	25 (32.9)	0.64

Data documented as n (%) or mean ± standard deviation. ST, Group ST (sternotomy); LIS, Group LIS (less invasive ~~approach~~); ICU, intensive care unit; LVAD, left ventricular assist device.

Supplementary Table 3 Comparison of perioperative vari~~lu~~ables between~~in~~ patients with PDD above or below~~lower than~~ the cut-off value (69.2mm) in LIS group

	PDD>69.2mm (n=16~~5~~)	PDD<69.2mm (n=54~~5~~)	P
Preoperative Impaired RV function, n (%)	10 (62.5~~66.7~~)	14 (25.9~~5~~)	<0.01
Postoperative RV failure, n (%)	3 (18.8~~20.0~~)	4 (7.4~~3~~)	0.19~~6~~
Postoperative RVAD, n (%)	3 (18.8~~20.0~~)	1 (1.9~~8~~)	0.04~~3~~
Postoperative Dialysis, n (%)	7 (43.8~~46.7~~)	10 (18.5~~2~~)	0.05~~3~~
Stroke, n (%)	5 (31.3~~33.3~~)	5 (9.3~~1~~)	0.04~~3~~
Sepsis, n (%)	4 (25.0~~26.7~~)	5 (9.3~~1~~)	0.11~~09~~
Acute respiratory distress syndrome, n (%)	1 (6.3~~7~~)	1 (1.9~~8~~)	0.41~~39~~
Tracheostomy, n (%)	5 (31.3~~33.3~~)	6 (11.1~~10.9~~)	0.07~~5~~
Pump thrombosis, n (%)	0 (0.0)	3 (5.6~~5~~)	0.45~~8~~

Data documented as n (%). PDD, pump diaphragm depth, RV; right ventricle, RVAD; right ventricular assist device.
